# Adaptation of the African couples HIV testing and counseling model for men who have sex with men in the United States: an application of the ADAPT-ITT framework

**DOI:** 10.1186/2193-1801-3-249

**Published:** 2014-05-16

**Authors:** Patrick S Sullivan, Rob Stephenson, Beau Grazter, Gina Wingood, Ralph Diclemente, Susan Allen, Colleen Hoff, Laura Salazar, Lamont Scales, Jeanne Montgomery, Ann Schwartz, Jasper Barnes, Kristina Grabbe

**Affiliations:** 1 Department of Epidemiology, Emory University Rollins School of Public Health, 1518 Clifton Road NE, Atlanta, GA 30322 USA; 2 Hubert Department of Global Health, Emory University Rollins School of Public Health, 1518 Clifton Road NE, Atlanta, GA 30322 USA; 3 Howard Brown Health Center, 4025 N Sheridan Rd, Chicago, IL 60613 USA; 4 Department of Behavioral Sciences and Health Education, Emory University Rollins School of Public Health, 1518 Clifton Road NE, Atlanta, GA 30322 USA; 5 Department of Pathology and Laboratory Medicine, Emory University School of Medicine, 1364 Clifton Road, Atlanta, GA 30322 USA; 6 Center for Research and Education on Gender and Sexuality, San Francisco State University, 835 Market Street, Suites 523-525, San Francisco, CA 94103 USA; 7 Institute for Public Health, Georgia State University, One Park Place, Suite 700, 30303 Atlanta, GA USA; 8 Division of HIV/AIDS Prevention, Centers for Disease Control and Prevention, 1600 Clifton Road NE, Atlanta, GA 30333 USA; 9 Licensed Family and Martial Therapist, 2200 Century Pkwy NE Suite 200, Atlanta, GA 30345 USA; 10 Center for Health and Behavioral Training, University of Rochester Medical Center, Rochester, NY USA

**Keywords:** HIV, AIDS, Prevention, Testing, Counseling, MSM, Couples, Adaptation, ADAPT-ITT

## Abstract

To respond to the need for new HIV prevention services for men who have sex with men (MSM) in the United States, and to respond to new data on the key role of main partnerships in US MSM epidemics, we sought to develop a new service for joint HIV testing of male couples. We used the ADAPT-ITT framework to guide our work. From May 2009 to July 2013, a multiphase process was undertaken to identify an appropriate service as the basis for adaptation, collect data to inform the adaptation, adapt the testing service, develop training materials, test the adapted service, and scale up and evaluate the initial version of the service. We chose to base our adaptation on an African couples HIV testing service that was developed in the 1980s and has been widely disseminated in low- and middle-income countries. Our adaptation was informed by qualitative data collections from MSM and HIV counselors, multiple online surveys of MSM, information gathering from key stakeholders, and theater testing of the adapted service with MSM and HIV counselors. Results of initial testing indicate that the adapted service is highly acceptable to MSM and to HIV counselors, that there are no evident harms (e.g., intimate partner violence, relationship dissolution) associated with the service, and that the service identifies a substantial number of HIV serodiscordant male couples. The story of the development and scale-up of the adapted service illustrates how multiple public and foundation funding sources can collaborate to bring a prevention adaptation from concept to public health application, touching on research, program evaluation, implementation science, and public health program delivery. The result of this process is an adapted couples HIV testing approach, with training materials and handoff from academic partners to public health for assessment of effectiveness and consideration of the potential benefits of implementation; further work is needed to optimally adapt the African couples testing service for use with male–female couples in the United States.

**Electronic supplementary material:**

The online version of this article (doi:10.1186/2193-1801-3-249) contains supplementary material, which is available to authorized users.

## Background

Since the earliest reports of AIDS in the United States, men who have sex with men (MSM) have been, and continue to be, the most adversely affected risk group in the US HIV epidemic (Sullivan and Wolitski 2007). Male couples represent a high-priority group for HIV prevention interventions, because primary partners have been identified as the source of approximately one-third (Goodreau et al. 2012) to two-thirds (Sullivan et al. 2009) of HIV infections among MSM. Many MSM have high rates of sexual risk behavior for HIV with main and casual partners. Potentially risky episodes with casual partners are often not disclosed to main partners (Gomez et al. 2012; Hoff et al. 2010; Gass et al. 2012). Each of these factors highlights the need for targeted HIV prevention services for male couples. The Office of the Global AIDS Coordinator, through dissemination of prevention guidelines for MSM in countries supported by the President’s Emergency Plan for AIDS Relief (PEPFAR), has recommended couples testing for male couples based on the strength of evidence from observational studies of heterosexual couples (PEPFAR 2011).

HIV testing is an important component of the National AIDS Strategy (The White House Office of National AIDS Policy 2010), but reported rates of HIV testing among partnered MSM are low (Chakravarty et al. 2012; Mitchell and Petroll 2012; Phillips et al. 2012). Among a large cohort of male couples, only 23% of men who had unprotected anal intercourse with an outside partner of HIV-positive or unknown status reported testing for HIV in the past 3 months (Chakravarty et al. 2012). In the same sample, 47% of those who had not tested for HIV in the past 6 months chose the response “*I am in a relationship*” as the reason for their not having tested in the past year, although less than half of these couples reported that their relationship was monogamous (Chakravarty et al. 2012). Clearly, innovative approaches are needed to increase rates of testing for male couples, to increase the success of primary prevention and to improve the rates of early identification of new HIV infection and early treatment engagement (Sullivan et al. 2012).

Couples HIV Testing and Counseling (CHTC) has been used as an HIV prevention strategy in Africa for over 20 years and is considered by the US Centers for Disease Control and Prevention (CDC) to be a “high leverage HIV prevention intervention” (Painter 2001). The critical difference between the CHTC model and the conventional model of individual testing or Voluntary HIV Counseling and Testing is that CHTC provides HIV testing and counseling to the couple: Couples receive different HIV counseling and prevention messaging based on the characteristics of their relationships and their joint HIV status. CHTC has demonstrated success in reducing sexual risk behavior and promoting mutual disclosure of HIV status among heterosexual serodiscordant couples (i.e., couples in which one is HIV negative and one is HIV positive) (Allen et al. 2003; Allen et al. 1992). In a non-randomized prospective study of heterosexual couples, HIV-negative women whose male partners had not participated in CHTC had a small reduction in HIV incidence, from 4.1/100 person-years (PY) to 3.4/100 PY. In contrast, the HIV incidence rate among women whose partners had participated in CHTC was about half as high (1.8/100 PY, p<0.04) (Allen et al. 1992). Previous studies have also demonstrated CHTC to be effective in increasing and sustaining condom use within primary couples and reducing sexual risk-taking within heterosexual serodiscordant couples who receive CHTC (Allen et al. 2003; Allen et al. 1992; Painter 2001; Roth et al. 2001). CHTC has received significant support through PEPFAR, has been adopted widely in sub-Saharan African countries with high adult HIV prevalence, and has been recommended by the World Health Organization as part of an integrated testing and biomedical prevention strategy (World Health Organization 2012).

The ADAPT-ITT framework, developed by Wingood and DiClemente (2008), provides a guide for systematically adapting HIV prevention interventions with proven efficacy to new target audiences. The framework grew from recognition of a lack of theoretical frameworks available for the adaptation of evidence-based interventions (EBIs). ADAPT-ITT consists of 8 sequential phases that offer HIV prevention providers and researchers a prescriptive method for adapting EBIs. It has been applied successfully to the adaptation of several HIV prevention interventions, including those for incarcerated populations (Latham et al. 2010), faith-based HIV interventions (Wingood et al. 2011b) and a community-recruited sample of Latina women (Wingood et al. 2011a). This report describes the use of the ADAPT-ITT framework to modify the original CHTC approach for use with male couples in the United States. We also discuss our critical path from concept to scale-up of the adapted intervention, which was different than the traditional approach to intervention development and testing.

## Methods and results

We applied the ADAPT-ITT framework using a mixed-methods approach: qualitative methods, quantitative surveys, key informant interviews, theater testing, a randomized prevention study, and an expanded program evaluation. Throughout the process, multiple funders, including government and private sector foundation funders, played key roles in supporting the adaptation. Our process followed the phases of adaptation specified in the ADAPT-ITT framework (Wingood and DiClemente 2008).

### Phase 1: assessment

The assessment phase typically involves data collection (focus groups, interviews and surveys) with members of the target population and target service providers, to determine the prevention needs of the risk population and to assess the capacity of an agency to adapt an intervention and implement it.

### Data collection

We conducted 4 focus groups with a total of 39 MSM in Atlanta, Chicago, and Seattle between September and October of 2009 (Stephenson et al. 2011). We conducted 2 online surveys of a total of 6,640 MSM to assess willingness to use a couples’ testing service and reasons for willingness or lack of willingness to use such a service. We conducted key informant interviews with staff from 2 community-based organizations (CBOs) that provide HIV testing services to MSM. We also incorporated feedback from health department staff in the City of Chicago, the State of Georgia, and the District of Columbia. Trainings were conducted for health department staff in the District of Columbia and City of Chicago, and trainers received feedback from participants about the trainings’ content. We received critical feedback from an NIH study section that reviewed an initial submission of an R34 proposal. Finally, we conducted a site visit to a CHTC provider in Lusaka, Zambia, with representatives of 2 community-based organizations (LS and BG) and a licensed marriage and family therapist (JM), and recorded their their observations about provision of the African CHTC service.

### Major findings

The results of focus groups with MSM have been previously reported (Stephenson et al. 2011). Major findings that influenced the assessment phase were an overall enthusiasm for using CHTC, and some important misconceptions about couples testing. The common misconceptions were that couples testing violated privacy laws, and that a counselor would elicit individual sexual histories from both partners in the joint CHTC session. HIV-positive men also reported using individual HIV testing as a way to disclose their HIV status to new sex partners. HIV-negative men across groups stated that they would use CHTC to learn one another’s status, so that they could discontinue condom use with their main partner if both were HIV-negative.

The results of the online internet survey relating to willingness to use CHTC have also been previously reported (Wagenaar et al. 2012). Major findings that influenced the assessment phase were a high (82%) overall intention of MSM to test with a male sex partner in the coming year (conditioned on the availability of a CHTC service in the United States); there was higher intention to use CHTC among men of color, younger men, and men with main sex partners. Main reasons for wanting to use CHTC among those who intended to use the service were learning one another’s HIV status, supporting their partner, and strengthening the relationship. Main reasons for not wanting to use CHTC among those who did not intend to use the service were wanting to learn one’s own status before testing with a partner, concern about a counselor asking questions about sexual history in front of the partner, and fear of being HIV-positive.

The results of a separate online survey reporting the prevalence and nature of sexual agreements have also been recently reported (Gass et al. 2012). The principal findings of these analyses were that agreements about whether or not outside sexual partners are allowed are nearly ubiquitous (91% of men with a main partner had some kind of agreement). Most of these agreements (64% of men) were agreements of monogamy, but over a quarter of men reported agreements that allowed outside sex partners with (24%) or without (3%) conditions. About a sixth of men reported breaking their agreement, and of those, over three-quarters of men did not tell their partner about breaking the agreement right away(Gass et al. 2012).

Feedback from community-based organization (CBO) staff and health department staff indicated some key concerns about implementing a couples testing service. First, both CBO and health department staff felt that a CHTC service would be feasible to implement only if it were time-neutral, i.e., if the time required were approximately the length of 2 individual voluntary counseling and testing sessions. Second, both groups were concerned about clarifying state laws regarding testing of partners together and developing appropriate models of informed consent. Other considerations raised by these groups were the need to consider service flow within service provision settings and how data elements for administrative reporting of provision of testing services would be provided. Participants in the training using the African training materials suggested removing some language that was perceived as value-laden, especially the recommendations to couples for monogamy and recommendations for disclosing HIV status to clergy.

An NIH study section raised concerns that intimate partner violence (IPV) was a possible negative outcome of the service and asked that plans be developed to evaluate this concern. CBO representatives and the licensed marriage and family therapist who observed the service provided in Africa expressed concerns that the group-informed consent and group delivery of pre-test information used in that program would be inappropriate for use in the United States.

### Modifications

Based on the findings in the Assessment phase, we made a number of modifications to the existing training materials, service flow, and marketing plan. First, our overall finding revealing a high intention to use the service encouraged us to continue the adaptation. The specific motivations to use or not use a CHTC service informed “frequently asked questions” for a public website (http://www.testingtogether.org), which refuted misconceptions about confidentiality and elicitation of sexual histories as part of the CHTC session.

The data on the high prevalence of agreements and breaking of agreements, coupled with men’s intention to discontinue condom use if both were HIV-negative, led us to add a new component to the CHTC service. Our reasoning was as follows: if a couple received concordant HIV-negative results and decided to discontinue condom use, they could actually be at increased risk of HIV transmission if one partner was exposed to HIV outside the relationship and came back into a relationship where condom use had been discontinued. Therefore, we felt it important to add an element to the CHTC service that established the existing agreement about outside partners, and provided skill-building to the couple around how to disclose violations of the agreement should they occur.

Based on the feedback from CBO providers, we aimed to limit the length of a CHTC session to no more than 45 minutes, the typical duration of 2 individual HIV testing sessions in many CBOs at the time. This duration was consistent with the length of service observed in many African couples testing programs. We also decided that consent and pre-test counseling should be conducted with the couple privately, rather than using the model of group consent and pre-test information observed in Africa.

### Phase 2: decision

The decision phase involves identifying candidate interventions and deciding whether to adopt or adapt the chosen intervention. ADAPT-ITT proposes a relatively narrow criterion for selection, identifying “starting point” EBIs for adaptation and specifying that an EBI should be selected through review of articles and publications written by CDC.

### Data collection

We used data from the focus group discussions (FGDs) described above, data from the internet survey of men in main partnerships described above, and information gathering from African couples testing trainers. We also systematically reviewed the HIV prevention literature to identify alternative couples HIV testing models that were not represented in CDC’s inventory of EBIs.

## Conclusions

We chose to work with the existing African couples HIV testing and counseling approach because there was no CDC-endorsed HIV testing or counseling EBI for male couples on http://www.effectiveinterventions.org, because of the robust training materials available for the African couples’ approach, and because of the substantial evidence for the prevention value of the African couples’ approach. CDC’s African CHTC training curriculum was developed with a broad range of academic and community stakeholders, is promoted and disseminated by CDC, and has been used extensively in Africa and Asia for nearly a decade in its current form. Although CDC had not formally reviewed the evidence base for CHTC in the US, CDC had stated that CHTC was a “high leverage HIV prevention intervention” in the African context (Painter 2001).

Despite the strengths of the CDC CHTC curriculum, we decided that adaptation was more appropriate than adoption. The primary reasons for this judgment were (1) the need to add a service component to address sexual agreements and (2) the need to make many of the examples and role-plays more culturally relevant to male couples than those in the original materials.

### Phase 3: adaptation

The adaptation phase involves pre-testing the intervention with the target audience to examine attitudes towards the format and content of the intervention and to receive feedback and recommendations for improving the acceptability of the intervention among the target audience. This is classically done with the original intervention; in our implementation we pre-tested CHTC with some modifications, such as with the use of non-group consent and with the addition of the agreements discussion.

### Data collection

We conducted theater testing-based FGD with MSM, HIV counselors, and clinic managers in Atlanta and Chicago. Theater testing is a pre-testing methodology adapted from social marketing. To facilitate an accurate assessment of reactions to the service, videos of the service are shown to groups of the intended audience and practitioners. Participants view an example of how the service is delivered to and experienced by the target population. In total 8 theater testing focus groups were conducted: 4 in Atlanta and 4 in Chicago. In each city, an FGD was conducted with each of 4 groups: black/African American MSM, white MSM, HIV counselors, and HIV testing clinic managers. Men in the MSM focus groups did not attend as couples, and HIV status was not an eligibility criterion; in most MSM groups, one or more men identified themselves as living with HIV during the discussion. Each FGD followed the same format. Participants were shown a video detailing the entire CHTC process, including the couple arriving at the testing site, signing consent forms, and receiving the CHTC service. Three separate endings were shown, illustrating the 3 possible HIV results that could be delivered in a CHTC session (serodiscordant, concordant negative, concordant positive). The films were stopped periodically to allow the participants to provide feedback on each stage of the service. At the end of the film participants provided feedback and recommendations on the service and discussed their willingness to use the service.

### Major findings

Table 1 illustrates key findings from the theater testing FGD. In agreement with the survey data presented for Phase 1, there was universally high acceptance and willingness to utilize CHTC among MSM in all groups. Providers and Clinic Managers also reported high levels of willingness to provide CHTC, and no supply-side barriers (e.g., limited space or counselor capacity) were identified to the successful integration of CHTC into existing testing sites. Participants in all groups reported that they were not aware of the availability of CHTC in their locales (CHTC was not currently available in either city at the time of the focus group discussions). The video showed a couple being screened for eligibility for CHTC: they were ineligible for CHTC if they had not been together for at least 3 months, if either of them reported recent (<12 months) IPV or if either reported feeling coerced to attend CHTC. All participants felt that the screening for IPV and coercion were important and reacted favorably to clients completing their intake and consent forms separately. However, participants in all groups did not agree with the 3-month relationship duration eligibility criterion; participants felt that this would prevent couples from using the service before they had sex, and would reinforce stigma against MSM by suggesting that early stage relationships were not “valid”.

**Table 1:** **Summary of results of theater testing of an adapted couples HIV testing service, by focus group participant type, Atlanta, Chicago and Seattle, October 2010**

Unit of analysis	Black MSM	White MSM	HIV counselors	HIV testing clinic managers
**Number of FGD**	2	2	2	2
**Total # in FGD**	18	19	17	14
**Have similar services been offered?**	No	No	No	No
**Should couple meet 3-month history criteria?**	No: MSM want to be able to come in when they’re pre-sexual, too.	No: MSM want to be able to come in when they’re pre-sexual, too.	No: No time limit should be placed on couple history so that new couples can feel protected, too.	No: They said this reinforces stigma and increases risky behavior
**Opinions on sign-in process & consent process**	Entry-forms and consent forms should be completed separately	Entry-forms and consent forms should be completed separately	Entry-forms and consent forms should be completed separately	Entry-forms and consent forms should be completed separately
**Opinions on service overview**	Opt-out clause should only be given once	Ground rules need to be made clear	Opt-out and ground rules good	Opt-out and ground rules good
	Ground rules are good	Should remind couple that they will hear each other’s results right before delivering them	Need to remind couples of confidentiality throughout session	Need to remind couples of confidentiality throughout session
**Opinions on agreement counseling**	Role-play is good	History of service is not relevant	Role-play is good and feel able to facilitate	Role-play is good and feel able to facilitate
		Role-play is good		
**Opinions on delivery of test results**	Should be delivered verbally	Should be delivered verbally	Should be delivered verbally	Should be delivered verbally
	Positive should always be delivered first if sero-discordant	Positive should always be delivered first if sero-discordant	Positive should always be delivered first if sero-discordant	Positive should always be delivered first if sero-discordant
	Should tell people “results are the same” before delivering results	Should tell people “results are the same” before delivering results	Should tell people “results are the same” before delivering results	Should tell people “results are the same” before delivering results
**Recommendations**	CVCT is needed	The service is essential	CVCT is needed	CVCT is needed
	Only male counselors should be used	Need facilities where couple can have privacy	Men’s clinic or after-hours clinic would be best	Male counselors should be used
	History of service is not relevant	History of service is not relevant		

In terms of the flow of the CHTC service, MSM participants felt that reminding the couple of the need for confidentiality at the beginning of the counseling session, establishing clear ground rules, and reminding the couple that they would hear each other’s results immediately before the delivery of results would improve comfort with the service.

In the video of a CHTC session, couples talked about their sexual agreements and role-played disclosure of breaking the agreements. Participants in all groups responded well to this element of the service, noting that it was an opportunity for couples to discuss the realities of sex and HIV in their relationships. Importantly, MSM reported they would be willing to participate in this element of the service and providers felt able to facilitate these discussions. In terms of how results should be delivered in a CHTC session (i.e., verbally, or on a written form, as is done in some Africa settings), all participants felt that results should be delivered verbally, and that the positive individual in a serodiscordant couple should be told first. For concordant results, participants liked the phrase “*your results are the same*” as a precursor to the delivery of the results.

### Modifications

Several minor modifications to the CHTC service were made as a result of the theater testing. The requirement for couples to be in a relationship for 3 months was dropped; instead, instructions for the delivery of CHTC were changed to state that it was at the discretion of the individual organization to establish relationship duration criteria, but that the service was recommended for couples at all relationship durations. In the original African service, the session opened with a description of the benefits of CHTC that included a history of CHTC; most participants reacted negatively to this and it was subsequently removed from the pre-testing steps of the service. Recommendations that the results be delivered verbally (to the positive individual first) and language around the delivery of concordant results were incorporated.

Other suggestions were not adopted. For example, several MSM and provider participants suggested that only male counselors should deliver the service. This recommendation was not taken as it was not universally reported by MSM and providers, and there was no precedent for it in the delivery of individual HIV testing and counseling. Restricting the delivery to male providers would also place an unnecessary burden on HIV testing sites already working with limited resources and skilled female counselors.

### Phase 4: production

In the production phase — i.e., the production of the service and related training materials — authors must balance the need to maintain fidelity to the core elements, underlying theory, and internal logic of the original approach with the realities of service delivery, which may include assessing the capacity of testing sites to provide services and the resources available for successful service delivery and integration. This phase also draws upon the results of the Assessment and Adaptation phases. Wingood and DiClemente (2008) note that authors need to decide whether the goal of adaptation is to produce a successfully adapted intervention for a new target population or to test whether the adapted intervention produces changes in theoretically important HIV prevention mediators and behavioral outcomes. For CHTC the aim was to produce a successfully adapted service for a new target population: adapting a previously successful couples’ testing model used with heterosexual couples in Africa for male-male couples in the US. In the Assessment phase we made changes to the content of the counseling messages (e.g., reduced emphasis on fertility), and these changes, as well as the changes identified by the target audience in the adaptation phase, were included in the service and related training materials.

The production process originally suggested by Wingood and DiClemente (2008) was used to guide Phase 4: a 7-stage plan for production is nested within this Phase. The information for stages 1–4 of the production phase drew upon information and decisions made earlier: (1) the aim of the adaptation: to provide couple’s HIV testing for male-male couples in the U.S; (2) the intervention (service) to be adapted: African CHTC; (3) the CDC publication citing the intervention as an EBI: for this we refer to the work of Painter et al., identifying CHTC as a “high leverage intervention”, and CDC’s dissemination of the training materials for the original African service; and (4) the new target population or context: male couples in the US.

Stage 5 identifies the core elements of the original intervention. These remained the same; the joint testing and counseling of male couples used the same protocol as used for heterosexual couples in Africa. Revisions to the training curriculum were contextual, removing discussions of fertility and religion. Most new material was produced in stages 6 and 7, which involved identifying the aim of the new materials and/or activities for inclusion in the adapted service and developing new materials and/or activities that may be more appropriate and relevant for the target population. In the assessment phase we had decided to include discussions of sexual agreements, given their relevance to male-male relationships, so we developed new content for the training materials. This content introduced the concept of sexual agreements, discussed the prevalence of typologies of sexual agreements, provided skills on counseling couples to form agreements and include them in prevention planning, and described activities that allowed providers to name types of agreements and practice counseling skills around agreement formation and disclosure of broken agreements. Other content included role-playing scenarios of male couples seeking CHTC, and an updated values-clarification exercise that included issues that were more contemporary and more pertinent to MSM, such as pre-exposure prophylaxis.

### Phases 5 & 6: topical experts and integration

The topical-experts phase involves collecting feedback from content experts on the first draft of the training materials and the flow and content of the service. The integration phase involves integrating that feedback into the adapted service and related training, resulting in a set of training materials suitable for pilot testing.

### Data collection

Content experts were identified in several key domains: HIV testing service delivery, couples counseling theory and practice, interventions for male-male couples, HIV risk-taking among MSM, scale development and data collection from couples, and IPV among male couples. Several of the content experts are authors on the current paper. The experts were engaged early in the adaptation process for a one-day meeting to review the preliminary materials and discuss changes. At the one-day meeting they were presented with the data from the assessment phase on willingness to use CHTC and the role of sexual agreements in male couples, and reviewed proposed changes to the service based on those data. A web-based survey of MSM in main partnerships was used to validate scales for measuring key elements of the theoretical framework.

### Modifications

The expert review resulted in a number of small changes made to the first draft of the materials. In addition to small changes in language and format, the most significant change was the proposal to change the name under which the service was marketed. It was felt that the “Couples” in CHTC would potentially result in self-censoring from the service for male partners who did not see themselves as a “couple”, with connotations of commitment and monogamy. Like the participants in the theater testing FGD, the content experts felt that CHTC should be available for all male partnerships, including those in which men were currently in, or intended to be in, a sexual relationship. As a result, it was recommended to market the service as “*Testing Together”.* We also noted the importance of training counselors explicitly about the use of language around identifying “couples”, to allow counselors to provide a high-quality service to men in different types of partnerships. Experts validated the incorporation of discussions and role playing around broken sexual agreements, and the licensed marriage and family therapist adapted the language and key counseling skills to be used at that stage. Experts also suggested scenarios for the role plays in the training materials.

After the expert review, scales were developed to capture domains of the Couples’ Interdependence Theory that forms the basis of the CHTC service (Lewis et al. 2006). The scales needed to be able to identify the impact of the service on the transformation of key elements of male-male relationships (e.g., communal coping and shared preferences). The development and validation of the scales is described elsewhere (Salazar et al. 2011); briefly, the purpose was to measure the perceived severity of HIV, preferences for sexual health outcomes, outcome and couple efficacy to avoid HIV, and communal coping strategies. Scale items were created based on theoretical definitions and results from 6 focus groups with MSM (recruited through the same venue-based sampling methodology used for FGD recruitment at other stages of the adaptation). Face and content validity of the scales were assessed with a panel of 6 content experts in the field of HIV prevention. Revised scales were subsequently administered to an online sample of 638 MSM who indicated being in a main partnership with another man for at least 3 months. All scales showed adequate reliability; evidence for construct validity was obtained for all scales except for perceived severity of HIV. The results indicated that these scales are reliable and valid measures that can be used in future HIV prevention research and practice with MSM couples (Salazar et al. 2011). The scales were used in Phase 8 (testing) as measures of the central concepts of change within couples resulting from exposure to the service. The scales have since also been used successfully in other studies examining IPV, HIV testing and relationship dynamics among male couples.

### Phase 7: training

The training process included training for current HIV counselors on how to deliver the CHTC service to male couples, and for trainers to train the counselors. The training of providers was based on the original CHTC training, which was a 4–5 day in-person didactic training. Our initial adaptation reduced the training to a 3-day in-person training, which topical experts felt was much more feasible and increased the likelihood of uptake among resource-constrained HIV testing organizations. It was decided that the audience for the training would be HIV counselors and testers, ideally with at least 6 months’ experience providing individual HIV counseling and testing.

**Figure 1:**
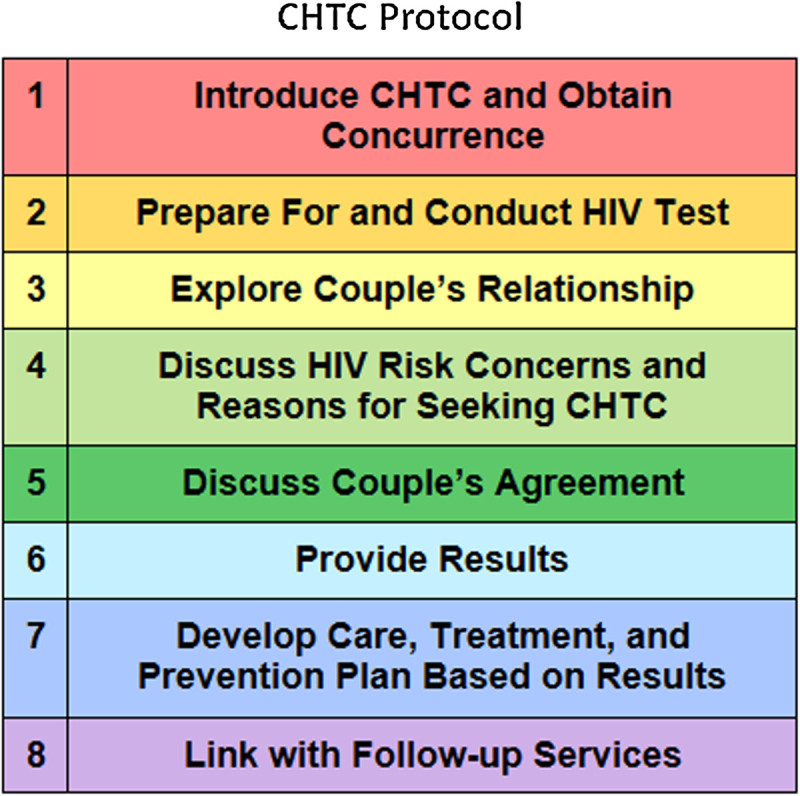
**Example of a training palm card with the CHTC protocol steps.** Materials such as these are used in training and support of counselors being trained to deliver the CHTC service.

The training materials consisted of 7 modules: background and discordance, introduction to couples counseling skills, initial session of CHTC, delivery of concordant negative results and prevention planning, delivery of concordant positive results and prevention planning, delivery of serodiscordant results and prevention planning, and implementation. Each module contained a combination of didactic teaching and participant activities aimed at highlighting key messages and building or reinforcing key skills. The materials placed a strong emphasis on developing counseling skills to enable the effective provision of CHTC; role plays allowed participants to practice simulated CHTC sessions and receive feedback from trainers. Several quality assurance efforts were also included: checklists of the protocol stages and their elements for counselors to use in delivering the service (see Figure 1 for an example), palm cards and posters of the protocol stages, and detailed instructions on the continued practicing of role plays and key questions to facilitate feedback after role plays. The implementation module was developed to address questions about demand generation, management of service provision within different organization types, organization-specific consent procedures, and a discussion of developing and implementing criteria for eligibility. The issue of eligibility was a frequent concern of trainees, and we approached this element by providing principles guiding the development of provider-specific criteria (i.e., the importance of mechanisms to exclude couples where one felt coerced, the importance of obtaining agreement on confidentiality and mutual disclosure, and the different types of couples that might seek CHTC services).

The first trainings were conducted in Atlanta and Chicago, the sites for the Stage 8 (Testing) pilot tests. Over the course of 4 years (2009–2013), more than 30 trainings were conducted throughout the US Additional trainings were conducted on request for community-based organizations and local health departments. In total, representatives from 73 testing sites were trained, representing 17 cities and 11 states. Over 300 HIV counselors and testers were trained in the provision of CHTC through mid-2013.

The large number of trainings conducted facilitated 2 key processes: the constant updating and refinement of the training materials, and the training of additional trainers. The training materials evolved significantly throughout this process. They came to include less content on the history of CHTC. They also included videos demonstrating key counseling skills and stages of the protocol, and training on consent and IPV/coercion screening procedures. At each revision the authors and key content experts reviewed the materials to ensure fidelity to the service. The materials were further refined by an expert in the production of training materials, focusing on reading level, comprehension, format and visual presentation.

Throughout this process other facilitators were trained to provide the CHTC training. Trainee facilitators first attended the training as participants, and then assisted with the delivery and feedback of participant activities, building up to delivering a single module, and eventually becoming a co-trainer. Trainings were provided by 2 co-trainers.

Another significant change to the CHTC training materials came when the service’s authors began working with Centers for Disease Control and Prevention — through the Division of HIV/AIDS Prevention — to include CHTC in the CDC list of effective HIV prevention strategies. At this point it was felt that a 3-day training might not be feasible for all testing sites. Thus the training was re-developed as a 2-day in-person training preceded by a 2-hour online webinar. Content about the history of and introduction to CHTC, key counseling skills, and an overview of the stages of the protocol were moved to the pre-training webinar. The 2-day in-person training remained focused on developing skills and practicing the delivery of the protocol. A further revision came with the transition from the webinar into a self-paced e-learning module, allowing the pre-training information to be viewed on the participant’s own schedule. The webinar and e-learning module were developed in collaboration with the Center for Health and Behavioral Training at the University of Rochester. At each stage of the training process, evaluation data were collected from training participants measuring attitudes towards the training format and content. In general, attitudes towards the trainings were positive, with favorable responses to the interactive nature of the training.

The finalized CHTC training materials can now be found at http://www.effectiveinterventions.org.

### Phase 8: testing

Our testing of the adapted intervention (service) comprised 2 studies: a phase IIa (exploratory, non-pivotal) randomized prevention trial of CHTC versus individual voluntary counseling and testing to assess acceptability and safety (IPV and relationship dissolution) in 1 site, and an expanded evaluation in 5 US cities to assess counselor satisfaction, IPV, relationship dissolution, and client satisfaction in a broader setting and with more counselors providing service. In both studies, we also assessed the prevalence of HIV serodiscordance among male couples as an indicator of possible program impact; based on African data, the clearest evidence of the preventive impact is with serodiscordant couples.

### Data collection

For the randomized prevention trial, we enrolled a total of 113 couples in an Atlanta community-based organization; couples with a recent history of IPV or where one or both partners felt coerced to test together were not randomized (Sullivan et al. 2014a). Other couples were randomized to either be tested for HIV together with the adapted approach, or to be tested separately. Regardless of study arm, couples were contacted at 3 months after their original service for retesting and a follow-up survey. Main outcomes were client satisfaction with their testing service, new IPV, and relationship dissolution after the testing service. For the extended evaluation, we trained an additional 7 sites, and additional counselors at the original study site. The additional sites included an academic medical center, a youth-oriented community-based organization, a city health department STI clinic, 2 LGBT health centers, and 2 additional community-based AIDS service organizations. In these sites, counselors were trained, and organizations were provided with iPod touch devices for counselor satisfaction surveys and with iPads for client satisfaction surveys administered immediately after the service was completed. This activity, which was considered to be a non-research evaluation project by Emory’s Institutional Review Board, also included an optional follow-up survey for couples 3 months after they received the testing service. The primary outcomes of the extended evaluation were: client satisfaction, counselor satisfaction, length of time required to deliver the service, 3-month post-service reporting of IPV and relationship dissolution by clients, and prevalence of HIV serodiscordance.

### Major findings

The results of the randomized prevention trial indicated that levels of satisfaction with the couples testing service were very high, and were not different from the levels of satisfaction with individual testing. (Sullivan et al. 2014a) There was no evidence of increased IPV or relationship dissolution after couples testing (p=0.60). In secondary exploratory analysis, no partner in a serodiscordant partnership reported unprotected anal intercourse within the discordant partnership in the 3 months after the service, regardless of study arm (Sullivan et al. 2014a). The prevalence of serodiscordance among 95 couples with testing data was 17% (Sullivan et al. 2014b).

In the extended evaluation, client and counselor satisfaction data from testing encounters with 365 couples depicted high levels of satisfaction, similar to what was observed in the RCT. This was a key observation because nearly all of the satisfaction data from the RCT related to the counseling services of 1 study counselor. Receiving equally positive reports from services provided by a larger and less experienced group of counselors makes this finding more robust. Across service settings in the expanded evaluation, we observed prevalence of serodiscordance ranging between 1 in 7 and 1 in 10 couples. About three-quarters of CHTC sessions were completed in 45 minutes or less.

## Conclusions

Based on a re-emergent HIV epidemic among US MSM (Prejean et al. 2011), evidence that main sex partners play a key role in that epidemic (Sullivan et al. 2009; Goodreau et al. 2012), the need for more effective prevention services (Sullivan et al. 2008), and a high observed prevalence of undiagnosed HIV infection (CDC 2006), we sought to develop a new HIV testing service for male couples in the United States. Guided by an ADAPT-ITT framework, we selected the African HIV couples testing service and applied a systematic, data-driven process to adapt and test the service. The outcome of this process is an adapted service that is acceptable to MSM and HIV prevention counselors and does not appear to cause any harms (e.g., IPV, relationship dissolution). The adapted service is available to the public health community for assessment of its effectiveness and consideration by policy makers of the potential benefits of its dissemination in the United States and elsewhere.

Although the ADAPT-ITT framework guided our process, we did depart from it. Importantly, ADAPT-ITT specifies that the base intervention should be selected from CDC-endorsed evidence-based interventions (Wingood and DiClemente 2008). In this case, there was no CDC-reviewed evidence-based testing service for couples, so we cast a broader net to identify base prevention services. Also, we conceptualized our work as adapting an HIV testing *service*, in contrast to an HIV prevention *intervention*. This distinction is in line with CDC’s categorization of effective interventions (http://www.effectiveinterventions.org); HIV testing by itself has inherent value as a prevention service, and we hoped to use a couples approach to recruit more MSM to testing, to increase the extent of disclosure of HIV-positive test results, and to improve the impact of prevention planning by helping couples make joint plans informed by their mutual serostatus. Finally, we note that our adaptation was not as linear and ordered as the ADAPT-ITT framework proposes. Because we collected data when funding (from a variety of sources) was available, we had primary data before formally deciding on adaptation over adoption; we convened our expert panel somewhat earlier in the process than ADAPT-ITT suggests; and we phased out testing over a relatively long period of time.

Our critical path from concept to scale-up was also different than a classical approach to prevention intervention development. In this case, we first developed data on willingness of MSM to use a service and motivations for using it, and after initial adaptation we conducted a phase IIa (exploratory, non-pivotal) randomized study with primary endpoints of acceptability and safety. This trial was not powered for efficacy against behavioral endpoints or new HIV infection. At the time the data collection for the randomized study concluded, we consulted with key stakeholders at the National Institutes of Health, the CDC, and representatives of community-based organizations. Together we considered possible mechanisms for funding a larger, randomized prevention trial with behavioral and HIV infection endpoints, and expectations of what data would be needed to support CHTC either as a service or an intervention, based on the current CDC hierarchy of evidence (Centers for Disease Control and Prevention 2007). Based on these consultations, we were encouraged to consider the couples testing service as a mode of delivering HIV testing — i.e., a testing strategy. We decided to focus on preliminary data on seropositivity rates, prevalence of serodiscordance, acceptability and safety, the long history of the service in Africa (Painter 2001), and the urgent need for new prevention approaches for MSM (Sullivan et al. 2012) to make a case for scale up of a new prevention “service,” rather than pursuing a phase III efficacy trial for a new “intervention” against behavioral or HIV incidence endpoints. Importantly, couples testing is in line with CDC recommendations for at least annual HIV testing for MSM. Providing a diverse set of options for HIV testing congruent with the realities of men’s lives and with the data about the risks of transmission within main partnerships, furthers the goals of public health and early detection and treatment of HIV for men (The White House Office of National AIDS Policy 2010).

A strength of our process was the involvement of multiple stakeholders in the process of assessment, adaptation, and evaluation. As described below, multiple public and private funders contributed to the process. We also were fortunate to have input from different prevention stakeholders: MSM, HIV counselors, management of community-based organizations and health departments, federal prevention scientists, and foundation grantmakers all provided input and guidance into the adaptation process. Importantly, these collaborations allowed us to design a service that was likely to be implementable in the kinds of service settings where we hoped it would be used. For example, we tailored the length of the service based on CBO and health department input about how long a service would be practical in their settings, and we asked individual HIV prevention counselors which areas of training they would wish to have if they were going to be counseling couples.

**Figure 2:**
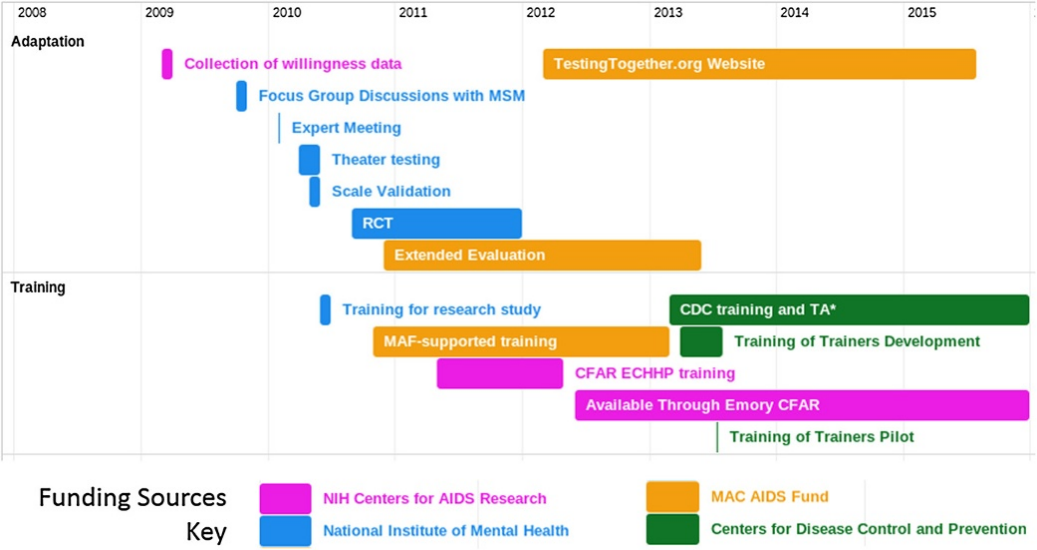
**Timeline for activities related to the adaptation and testing of a couples HIV testing service for by type of funding, United States, 2009–2015.** Emory CFAR: Emory Centers for AIDS Research; NIH/NIMH: National Institutes of Health/National Institute of Mental Health CDC: US Centers for Disease Control and Prevention; ECHPP: Enhanced Comprehensive HIV Prevention Planning Project; RCT: Randomized Clinical Trial.

This process of adaptation and testing is notable for the extent to which multiple funders, both public and private, supported different phases of the process (Figure 2). Pilot data about willingness of MSM to test with their partners were collected through an Emory Center for AIDS Research small-grant mechanism, which is designed to support junior investigators in developing preliminary data towards developing larger research agendas. The National Institutes of Mental Health supported the initial research through an intervention development (R34) mechanism, allowing the initial adaptation and data collection through the end of the randomized prevention study. At this point, the preliminary research was complete and the adapted service held promise, but there was not critical scientific mass, or extended evidence of more generalizable acceptability and relevance. At this point the MAC AIDS Fund provided a critical bridge between the end of the first phase of research and the transition into publicly-supported packaging and rollout. Specifically, this foundation funder supported extended evaluation, expanded training, and activities related to developing training materials jointly with CDC partners. This support allowed the new service to establish a broader base of credibility and implementation and to maintain momentum following the initial randomized trial. Ultimately, sustainability requires the involvement and support of a governmental leader and champion; in this case, CDC has provided leadership and in-kind contributions to the process of developing and finalizing training materials, and as of March 1, 2013 has taken over responsibility for the national training program in the United States.

There are a number of important next steps for the implementation of this prevention service. First, there is interest in the service, for both male couples and for male–female couples, from countries outside the United States. Each country considering this service must make an assessment of whether the service is appropriate as we have adapted it, or whether further adaptation is required. The ADAPT-ITT framework anticipates and provides a structure to answer these questions with an iterative process. On the other hand, some countries might want to pursue a different critical path and propose a more formal, randomized evaluation of the service against endpoints of possible harms, behavioral risks, and/or HIV or STI incidence outcomes. An easy initial step is to assess interest in the prevention service among male couples. For example, we have conducted a 7-country study of willingness to use CHTC among MSM in Australia, Brazil, Canada, South Africa, Thailand, the United Kingdom, and the United tates. The results indicated high intent to use a couples testing service among MSM in all countries, with a range of intent to use the service in the next year from 79-90% (Stephenson et al. 2013). In Canada and the United Kingdom we have participated in initial community meetings, where the idea of couples testing for male couples was discussed with physicians, public health practitioners, MSM, CBO staff, and laboratorians. These discussions are fruitful ways to bring forward the concerns and perspectives of stakeholders and to formulate a process to evaluate if and how couples testing might be implemented in a particular community.

Another challenge will be how to address issues of reimbursement for the CHTC service in different service settings. In many ways, this issue is analogous to the challenges of supporting individual HIV testing with prevention counseling. Currently, all CDC grantees who receive funds to support HIV testing are permitted and encouraged to use those funds to provide CHTC services. Costs of the service have not been estimated across a range of organizations, but it is estimated that they do not differ greatly from costs associated with testing individuals; in fact there may be cost-savings in some settings. Anticipated costs for CHTC include the cost of the testing devices, approximately one hour of counselor time (to include 30–45 minutes with the couple and recordkeeping), and the costs of registration, test reporting, and administrative costs. With respect to privately-funded healthcare providers, the laboratory costs of routine HIV screening are covered by health insurance plans for those aged 15–25 years in light of recent US Preventive Service Task Force recommendations (Moyer 2013). The additional costs of prevention counseling associated with HIV testing are not routinely reimbursable, but may be through some plans. In the future, partnerships could be considered in which community providers trained to provide CHTC could support medical providers by providing the CHTC service as a referred service in appropriate couples.

Finally, there are still scientific and evaluation opportunities to better understand the possible role of couples testing for HIV and prevention of sexually transmitted infections among male–female couples in the United States. Based on the strength of evidence and long history of service provision to male–female couples in Africa, CDC has developed an integrated training approach that prepares counselors to provide couples testing to any couple, regardless of sex of the couple members. However, it is likely that our understanding of the best ways to provide the couples testing service to male–female couples will evolve. For example, it is not clear whether the discussion of sexual agreements is as relevant for male–female couples, or whether the counselor initiating a discussion of agreements will be acceptable to male–female couples. Survey work about willingness to test with opposite-sex partners and qualitative studies of the intentions to use a couples testing service are needed. Incremental revisions to service delivery can be implemented as needed from the expanding base of evidence. Further, existing systems to monitor the provision of testing services (Centers for Disease Control and Prevention 2013) should be modified to allow monitoring of the uptake and outcomes of CHTC supported by federal resources. Finally, additional research is needed on ways in which CHTC can be used to leverage high impact prevention strategies, including pre-exposure prophylaxis (Grant et al. 2010; Baeten et al. 2012) and linkage to effective clinical care for persons living with HIV.

HIV couples testing is an African prevention service that has been adapted and brought to program in the United States. It is an example of South-to-North knowledge transfer, and its adaptation illustrates that some key principles of HIV epidemiology and prevention transcend specific risk and geographic settings. This couples HIV prevention service complements existing testing and counseling services, and we are hopeful that an increased number of HIV testing options for MSM will support greater uptake of routine HIV testing.

## Authors’ original submitted files for images

Below are the links to the authors’ original submitted files for images. Authors’ original file for figure 1 Authors’ original file for figure 2

## Abbreviations

CBO: Community-based organization
CDC: Centers for disease control and prevention
CHTC: Couples HIV testing and counseling
EBI: Evidence-based intervention
FGD: Focus group discussion
IPV: Intimate partner violence
LGBT: Lesbian, gay, bisexual, transgender
MSM: Men who have sex with men
PY: Person-years.
