# Erratum to: Adaptation of the African couples HIV testing and counseling model for men who have sex with men in the United States: an application of the ADAPT-ITT framework

**DOI:** 10.1186/2193-1801-3-396

**Published:** 2014-07-30

**Authors:** Patrick S Sullivan, Rob Stephenson, Beau Gratzer, Gina Wingood, Ralph Diclemente, Susan Allen, Colleen Hoff, Laura Salazar, Lamont Scales, Jeanne Montgomery, Ann Schwartz, Jasper Barnes, Kristina Grabbe

**Affiliations:** Department of Epidemiology, Emory University Rollins School of Public Health, 1518 Clifton Road NE, Atlanta, GA 30322 USA; Hubert Department of Global Health, Emory University Rollins School of Public Health, 1518 Clifton Road NE, Atlanta, GA 30322 USA; Howard Brown Health Center, 4025 N Sheridan Rd, Chicago, IL 60613 USA; Department of Behavioral Sciences and Health Education, Emory University Rollins School of Public Health, 1518 Clifton Road NE, Atlanta, GA 30322 USA; Department of Pathology and Laboratory Medicine, Emory University School of Medicine, 1364 Clifton Road, Atlanta, GA 30322 USA; Center for Research and Education on Gender and Sexuality, San Francisco State University, 835 Market Street, Suites 523-525, San Francisco, CA 94103 USA; Institute for Public Health, Georgia State University, One Park Place, Suite 700, Atlanta, GA 30303 USA; Division of HIV/AIDS Prevention, Centers for Disease Control and Prevention, 1600 Clifton Road NE, Atlanta, GA 30333 USA; Licensed Family and Martial Therapist, 2200 Century Pkwy NE Suite 200, Atlanta, GA 30345 USA; Center for Health and Behavioral Training, University of Rochester Medical Center, Rochester, NY USA

## Correction

The author list in the original version of this article (Sullivan et al. 2014) incorrectly listed the surname of Dr. Beau Gratzer as ‘Grazter’. The publisher would like to apologise to Dr. Gratzer for the error.
