# Supplementary figures and images for: Adaptation of the African couples HIV testing and counseling model for men who have sex with men in the United States: an application of the ADAPT-ITT framework
Source: Springerplus. 2014 May 16;3(1):249. doi: 10.1186/2193-1801-3-249 (PMC4035496; doi:10.1186/2193-1801-3-249)

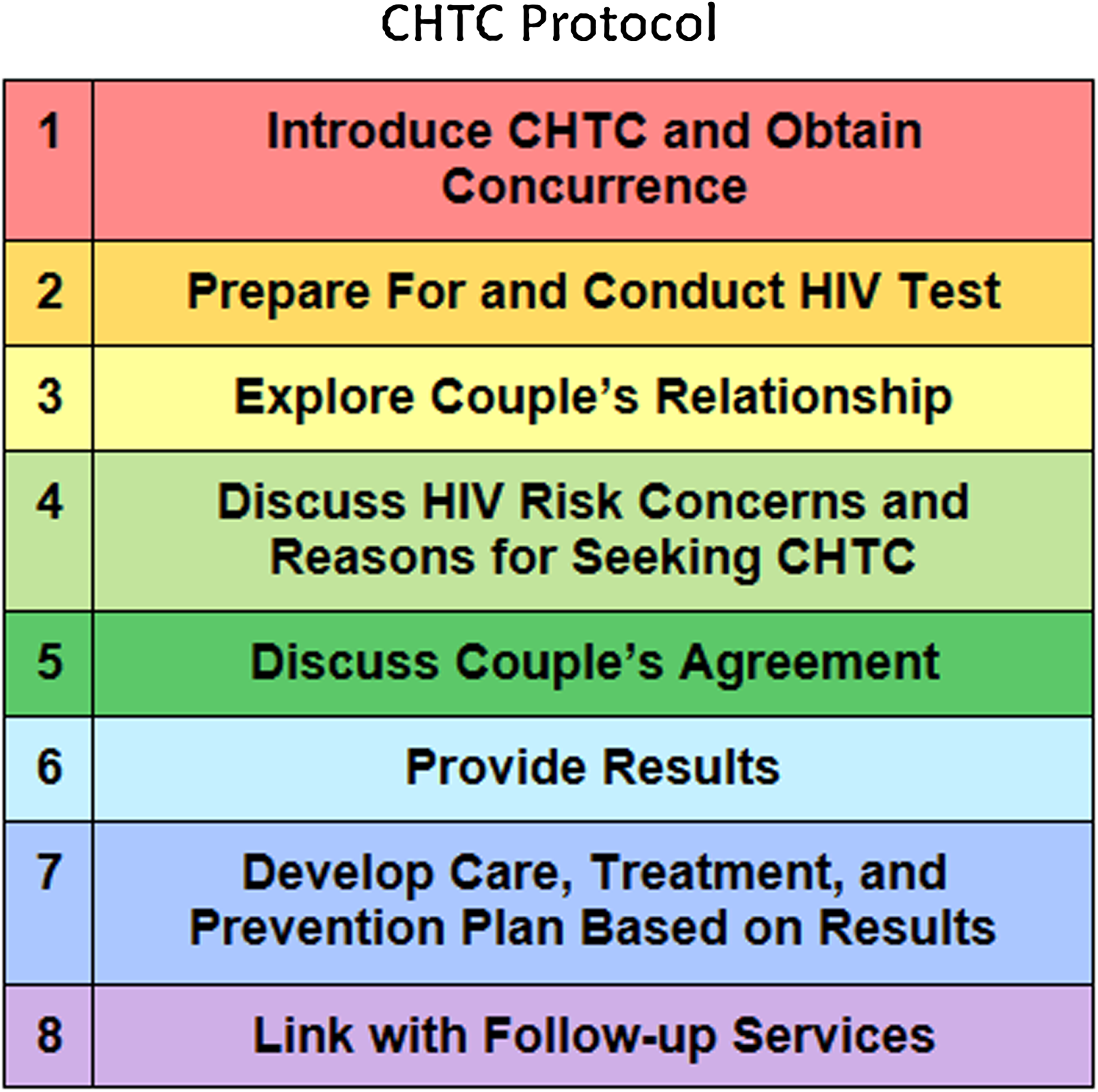

Supplement: Supplementary file 1 — Authors’ original file for figure 1 [file 40064_2014_965_MOESM1_ESM.tif]

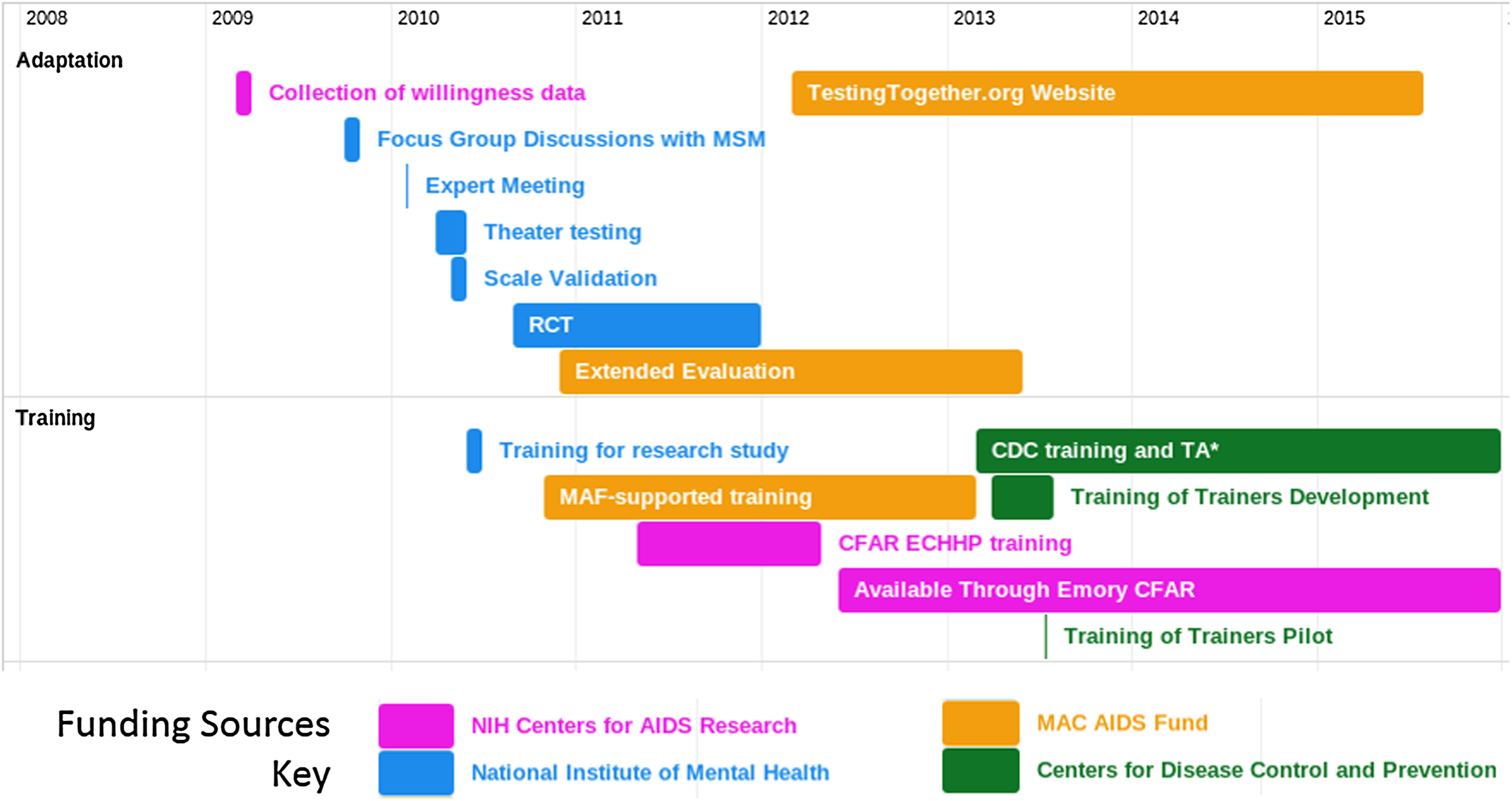

Supplement: Supplementary file 2 — Authors’ original file for figure 2 [file 40064_2014_965_MOESM2_ESM.tif]
